# Lack of Targetable FGFR2 Fusions in Endemic Fluke-Associated Cholangiocarcinoma

**DOI:** 10.1200/GO.20.00030

**Published:** 2020-04-21

**Authors:** Sarinya Kongpetch, Apinya Jusakul, Jing Quan Lim, Cedric Chuan Young Ng, Jason Yongsheng Chan, Vikneswari Rajasegaran, Tse Hui Lim, Kiat Hon Lim, Su Pin Choo, Simona Dima, Irinel Popescu, Dan G. Duda, Veerapol Kukongviriyapan, Narong Khuntikeo, Chawalit Pairojkul, Steven G. Rozen, Patrick Tan, Bin Tean Teh

**Affiliations:** ^1^ Cholangiocarcinoma Screening and Care Program and Cholangiocarcinoma Research Institute, Khon Kaen University, Khon Kaen, Thailand; ^2^ Department of Pharmacology, Khon Kaen University, Khon Kaen, Thailand; ^3^ The Centre for Research and Development of Medical Diagnostic Laboratories and Department of Clinical Immunology and Transfusion Sciences, Khon Kaen University, Khon Kaen, Thailand; ^4^ Oncology Academic Clinical Program, Duke-NUS Medical School, Singapore; ^5^ Lymphoma Genomic Translational Research Laboratory, Division of Cellular and Molecular Research, National Cancer Centre Singapore, Singapore; ^6^ Laboratory of Cancer Epigenome, Division of Medical Science, National Cancer Centre Singapore, Singapore; ^7^ Division of Medical Oncology, National Cancer Centre Singapore, Singapore; ^8^ Cytogenetics Laboratory, Department of Molecular Pathology, Singapore General Hospital, Singapore; ^9^ Department of Anatomical Pathology, Singapore General Hospital, Singapore; ^10^ Center of Digestive Diseases and Liver Transplantation, Fundeni Clinical Institute, Bucharest, Romania; ^11^ Edwin L. Steele Laboratories for Tumor Biology, Department of Radiation Oncology, Massachusetts General Hospital and Harvard Medical School, Boston, MA; ^12^ Department of Surgery, Khon Kaen University, Khon Kaen, Thailand; ^13^ Department of Pathology, Khon Kaen University, Khon Kaen, Thailand; ^14^ Program in Cancer and Stem Cell Biology, Duke-NUS Medical School, Singapore; ^15^ Centre for Computational Biology, Duke-NUS Medical School, Singapore; ^16^ SingHealth/Duke-NUS Institute of Precision Medicine, National Heart Centre, Singapore; ^17^ Cancer Science Institute of Singapore, National University of Singapore, Singapore; ^18^ Genome Institute of Singapore, Singapore; ^19^ Institute of Molecular and Cell Biology, Singapore

## Abstract

**PURPOSE:**

Cholangiocarcinoma (CCA) remains a disease with poor prognosis and limited therapeutic options. Identification of driver genetic alterations may lead to the discovery of more effective targeted therapies. CCAs harboring *FGFR2* fusions have recently demonstrated promising responses to FGFR inhibitors, highlighting their potential relevance as predictive biomarkers. CCA incidence is high in the northeast of Thailand and its neighboring countries because of chronic infection with the liver fluke *Opisthorchis viverrini* (Ov). However, there are currently no available data on the prevalence of *FGFR* alterations in fluke-associated CCA in endemic countries.

**MATERIALS AND METHODS:**

In this study, we performed anchored multiplex polymerase chain reaction target enrichment RNA sequencing of *FGFR1-3*, validated by fluorescence in situ hybridization and Sanger sequencing, in 121 Ov-associated and 95 non–Ov-associated CCA tumors.

**RESULTS:**

Compared with non–fluke-associated CCA (11/95; 11.6%), *FGFR2* fusions were significantly less common in fluke-associated CCA (1/121; 0.8%; *P* = .0006). All *FGFR* fusions were detected exclusively in intrahepatic CCAs and were mutually exclusive with *KRAS/ERBB2/BRAF/FGFR* mutations, pointing to their potential roles as oncogenic drivers.

**CONCLUSION:**

*FGFR2* fusions are rare in fluke-associated CCA, underscoring how distinct etiologies may affect molecular landscapes in tumors and highlighting the need to discover other actionable genomic alterations in endemic fluke-associated CCA.

## INTRODUCTION

Cholangiocarcinoma (CCA) is a biliary tract malignancy with limited treatment options and a poor 5-year survival rate of < 20% after surgery and chemotherapy.^1^ CCA can be classified into intrahepatic and extrahepatic (perihilar and distal) subtypes on the basis of anatomic location. Several risk factors for CCA are related to geography and etiology. For example, chronic infection with a liver fluke called *Opisthorchis viverrini* has been associated with CCA carcinogenesis in the northeast of Thailand and its neighboring countries, Laos and Cambodia. In contrast, primary sclerosing cholangitis is the most common risk factor for CCA in Western countries.^2^ Other risk factors include stones in the hepatobiliary ducts, congenital choledochal cysts, hepatitis viruses, inflammatory bowel disease, alcohol, smoking, and fatty liver disease.^3^ The molecular mechanisms underlying CCA tumorigenesis and heterogeneity remain poorly understood. Recently, technological advancements in genomic research, particularly next-generation sequencing (NGS) techniques, have accelerated the study of the molecular taxonomy of a spectrum of cancers and the discovery of novel genetic alterations contributing to tumorigenesis.^4-8^ Chromosomal rearrangements, particularly gene translocations that lead to oncogenic kinase activation, have been identified and validated as driver events in many cancer types. Such fusion kinases, which are considered to be druggable, may be ideal targets for antikinase therapy. In CCA, fibroblast growth factor receptor (*FGFR*) fusions have recently been identified and shown to be functionally relevant, contributing to tumorigenesis and progression.^9,10^ Subsequently, patients with *FGFR* genetic alterations were shown to respond more effectively to FGFR inhibitors compared with standard treatment.^11^ Therefore, an effective method to detect *FGFR* genetic alterations, which may serve as a companion biomarker, is needed. A recently developed technique called anchored multiplex polymerase chain reaction (AMP), which involves rapid target enrichment followed by NGS, has been demonstrated to be an efficient technique for detecting fusion genes,^12^ particularly in capturing unknown partner gene(s) of the fusion transcript by using a targeted RNA sequencing technology. In addition, it has robust detection capabilities for low-abundance fusion genes that fluorescence in situ hybridization (FISH) cannot detect.

**CONTEXT**

**Key Objective**
Are FGFR alterations prevalent in fluke-associated cholangiocarcinoma (CCA) in endemic countries?
**Knowledge Generated**
Fusions involving FGFR family genes, in particular FGFR2, were significantly enriched in non–fluke-associated CCA compared with fluke-associated cases. All FGFR fusion-positive CCA tumors were exclusively intrahepatic and mutually exclusive with somatic mutations in other kinase-related genes, including KRAS/ERBB2/BRAF/FGFR, implying their potential roles as cancer drivers.
**Relevance**
This study suggests that distinct etiologies may affect molecular landscapes in CCA and highlights the importance of conducting genomic studies on cancer in diverse populations.

FGFRs are transmembrane receptor proteins belonging to the receptor tyrosine kinase family and consist of four members: FGFR1, FGFR2, FGFR3, and FGFR4. Ligand-dependent dimerization, which forms a complex comprising two fibroblast growth factors (FGF), two FGFRs, and two heparin sulfate chains, leads to a conformational shift in the structure of the receptor that activates its intracellular kinase domain, resulting in intermolecular transphosphorylation of the tyrosine kinase domains and subsequent activation of intracellular downstream effectors such as Ras/MAPK, PI3K/AKT, STAT, and PLCγ.^13,14^ Alterations in *FGFR* genes, including activating mutations, chromosomal translocations, and gene amplifications, can result in ligand-independent signaling, which, in turn, leads to constitutive receptor activation. For example, chromosomal translocations can result in the fusion of the FGFR kinase domain to the dimerization domain of another protein, leading to constitutive kinase activation.^10^ Accumulating evidence indicates that *FGFR* alterations promote tumorigenesis by inducing mitogenic and survival signals as well as cancer progression by promoting epithelial-mesenchymal transition, invasion, and tumor angiogenesis.^13^ Therefore, FGFR inhibitors have recently been trialed in patients with CCA. At least two clinical studies showed the effect of single-agent FGFR inhibitors in patients with CCA harboring *FGFR2* fusions. In a multicenter, open-label, phase II study on BGJ398 in advanced or metastatic CCA with *FGFR* alterations, all responsive cases harbored *FGFR2* fusions. The overall response rate was 14.8%, and this response was even higher in the group harboring *FGFR2* fusion only (18.8%).^15^ In another study, inoperable intrahepatic CCAs harboring *FGFR2* gene fusions were further evaluated for the response to derazantinib (ARQ 087). This oral agent with potent pan-FGFR activity showed an overall response rate of 20.7% and disease control rate of 82.8%.^16^ Altogether, these clinical responses toward FGFR inhibitors suggest that certain *FGFR* alterations, particularly *FGFR2* fusions, may serve as biomarkers for personalized CCA therapy. Other *FGFR* alterations, such as point mutations and amplifications, have not shown obvious correlation, although larger cohort studies are needed.

To date, several cohort studies have detected frequent *FGFR* gene alterations (10%-40%) in non–fluke-associated CCA.^9-11,17^ However, the frequency of these alterations, especially *FGFR* fusion, in fluke-associated CCA remains unknown. Here, using AMP and targeted sequencing, we screened 216 CCA tumors from different geographic regions (121 fluke-associated CCAs from northeast Thailand *v* 95 non–fluke-associated CCAs from Romania and Singapore) for *FGFR* fusions.

## MATERIALS AND METHODS

### Ethics Approval and Consent to Participate

The study was performed in accordance with the Declaration of Helsinki. This study has been approved by the SingHealth Centralised Institutional Review Board (2006/449/B), the Ethics Committee of the Clinical Institute of Digestive Diseases and Liver Transplantation, Fundeni (215/18.01.2010), and the Human Research Ethics Committee at Khon Kaen University (HE471214). Primary tumor and matched normal samples (non-neoplastic liver or whole blood) were obtained from the SingHealth Tissue Repository (Singapore), the Fundeni Clinical Institute (Romania), and Khon Kaen University (Thailand), with signed informed consent.

### Clinical Specimens and Nucleic Acid Extraction for AMP

Clinicopathological information, including age, sex, and tumor subtype, was reviewed retrospectively. RNA (250 ng) extracted using the RNeasy Mini kit (Qiagen, Hilden, Germany) was subjected to library construction for AMP, as detailed in the Data Supplement. A pooled library (pool of 48 libraries) was quantified using quantitative polymerase chain reaction (PCR; Kapa Biosystems, Woburn, MA), then normalized and processed for sequencing on the MiSeq (Illumina, San Diego, CA) according to the manufacturers’ standard protocols. Sequencing was performed at the Duke-NUS Genome Biology Facility in Singapore.

### Data Analysis and Protein Prediction

The analysis of a single set of FASTQ files was run by the Archer Analysis Pipeline version 3.0 (ArcherDX, Boulder, CO; Data Supplement). The data that support the findings of this study are available from the corresponding author on reasonable request.

### Validation of FGFR Fusions

PCR was performed using fusion-specific primers and verified via Sanger sequencing. Each sequencing trace was aligned to the reference sequence using Lasergene 10.1 (DNASTAR, Madison, WI). To identify *FGFR2* rearrangements, break-apart FISH was performed on formalin-fixed paraffin-embedded (FFPE) tumors using hybridization probes. Expression of *FGFR2* transcripts was determined by quantitative real-time PCR. Experimental details are included in the Data Supplement.

### Copy-Number Analysis

To determine somatic copy number alterations, we used our published data of 175 cases with single-nucleotide polymorphism (SNP) array data.^5^ Briefly, raw SNP array data were processed using Illumina Genome Studio. ASCAT v2.0 was used to estimate allele-specific copy-number profiles.^18^ The regions of copy-number alteration were determined based on their relative copy number using the “copy-number” R package. A relative copy change of > 1.5 and < 0.7 are used as cutoffs for copy gain and copy loss, respectively.

### Statistical Analysis

The clinicopathological parameters were classified in categorical and continuous variables. Categorical variables were summarized as total counts and frequencies (%). Continuous variables were classified into two groups according to the median. Correlation between the presence of *FGFR* fusions and clinicopathological variables was performed with SPSS software (SPSS software v.19; IBM Corporation, Armonk, NY). Univariable comparisons of each variable by *FGFR* fusion status were assessed using χ^2^ and Fisher’s exact tests. Survival analysis was determined using the Kaplan-Meier method (GraphPad Prism 5; GraphPad Software, San Diego, CA).

## RESULTS

### Discovery of Novel *FGFR* Fusions in CCA Using AMP RNA Sequencing

**FIG 1:**
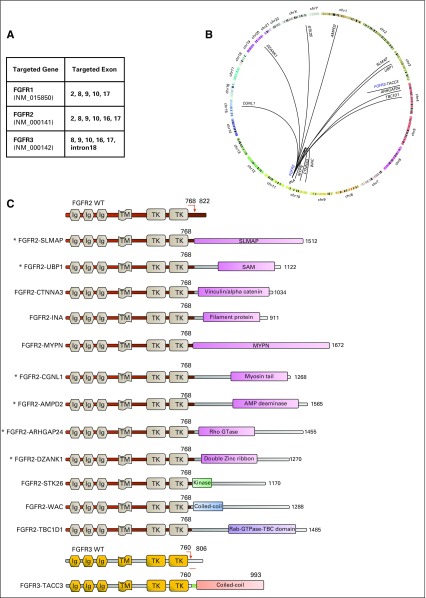
Discovery of *FGFR* fusions in cholangiocarcinoma (CCA). (A) Targeted exons of *FGFR* breakpoints identified by anchored multiplex polymerase chain reaction. (B) Circos plot showing chromosome locations of *FGFR* fusions. (C) All *FGFR* fusions found in our cohort of 216 cases of CCA. (*) Represents novel gene fusions that have not been previously reported. All are predicted to produce in-frame transcripts. The scheme also shows the predicted total number of amino acid residues for each chimeric fusion, calculated from the transcription start site of *FGFR* until the end of subsequent partners.

To identify *FGFR* fusions, we used AMP RNA sequencing, which is a targeted enrichment method that uses specific probes to capture exons of *FGFR1*, *FGFR2*, and *FGFR3* that are known to break and fuse with other partners (Fig 1A). We first validated the assay kit by using the fusion-positive urothelial cell line RT112, which is known to harbor the *FGFR3-TACC3* fusion.^19^ We were able to detect a fusion transcript in which exon 17 of *FGFR3* was fused with exon 11 of *TACC3* in a 5′ to 3′ direction with supporting reads of 11018 (Data Supplement).

Next, we performed AMP RNA sequencing on 216 CCA tumors, using the same probes as before. We identified 13 unique *FGFR* fusion products (12 to *FGFR2* and 1 to *FGFR3*). All the *FGFR* fusions were in-frame and had intact kinase domains with the ability to activate downstream kinases. Of the 13 *FGFR* fusions, 6 were novel. *FGFR2* is mapped to chromosome 10q26.1 (a known fragile site on chromosome 10), and the *FGFR2* fusion gene partners were mapped to chromosomes 1 (*AMPD2*), 3 (*SLMAP*, *UBP1*), 4 (*ARHGAP24*, *TBC1D1*), 10 (*CTNNA3*, *INA*, *MYPN*, *WAC*), 15 (*CGNL1*), 20 (*DZANK1*), and the X chromosome (*STK26*; Fig 1B and Data Supplement). In addition, *MYPN* and *INA*, which are located on the forward strand of chromosome 10 at 10q21.3 and 10q24.33, respectively, were in the opposite orientation from *FGFR2*, which is located on the reverse strand, indicating that the fusion genes were generated by intrachromosomal inversion. Besides the 12 *FGFR2* fusions, a single *FGFR3-TACC3* fusion product was detected in a fluke-associated CCA tumor. *FGFR1* fusion events were not detected in our cohort. *FGFR3-TACC3*, *FGFR2-STK26*, *FGFR2-WAC*, and *FGFR2-TBC1D1* (Figs 1B and 1C; Table 1) were also previously detected by whole-genome sequencing.^5^

**TABLE 1:**
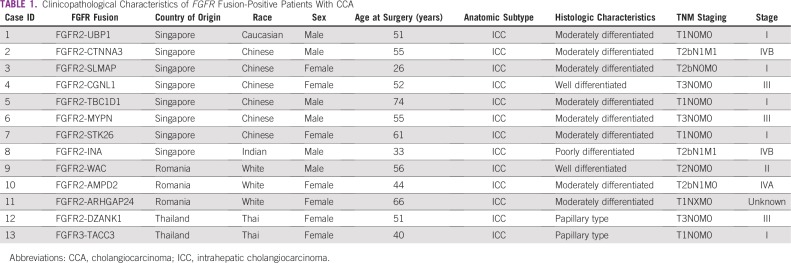
Clinicopathological Characteristics of *FGFR* Fusion-Positive Patients With CCA

Interestingly, 9 *FGFR2* fusions consisted of the in-frame fusion of the *FGFR2* amino terminus (exons 1-17) and the carboxyl terminus of unique 3′ partners, including *AMPD2* (exons 1-19), *SLMAP* (exons 2-23), *UBP1* (exons 6-16), *ARHGAP24* (exons 3-10), *CTNNA3* (exons 14-18), *INA* (exons 2-3), *MYPN* (exons 6-24), *CGNL1* (exons 9-19), and *DZANK1* (exons 9-21). Schematics of the chimeric fusion proteins, with protein domains and predicted lengths, are shown in Figure 1C. *UBP1*, which contains a sterile alpha motif (SAM) at amino acid residues 348 to 426, and *CGNL1*, which contains coiled-coil domains, have both been previously reported to play a role in protein interaction and dimerization.^10^ *AMPD2* encodes adenosine monophosphate deaminase 2, which is involved in purine metabolism by converting AMP to IMP.

*CGNL1* and *ARHGAP24* are both involved in the regulation of small GTPase proteins involved in adherent and tight cell-cell junctions and G-protein coupled receptor signaling. *CGNL1* (encoding cingulin-like 1), which is predicted to form a coiled-coil dimer, was previously reported to be involved in a chromosomal inversion (15q21.2;q21.3), placing it upstream of the CYP19 coding region in patients with aromatase excess syndrome, an autosomal dominant disorder characterized by increased extraglandular aromatization of steroids.^20^ Finally, *ARHGAP24*, which encodes Rho GTPase activating protein 24, has been found to regulate neuronal growth and is reported to be deleted (4q21.23;q21.3) in patients with autism spectrum disorders.^21^ Recently, two fusions that have been detected in our cohort, *FGFR2-INA* and *FGFR2-CTNNA3*, were reported in mixed neuronal-glial tumors (MNGTs). These FGFR2 fusions have been functionally characterized in vitro. They mediated the oncogenic signaling and growth via MAPK and PI3K/mTOR pathway activation in MNGTs.^22,23^

**FIG 2:**
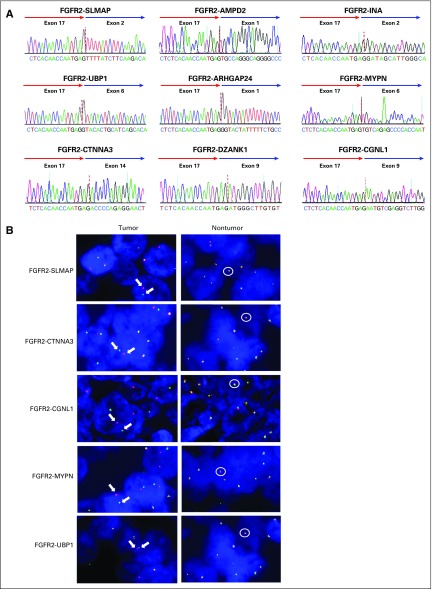
*FGFR2* and its fusion partners. (A) Schematic representation of the identified mRNA fusion gene. *FGFR2* fusion genes are represented. Sanger sequencing confirmed the chimeric junction between *FGFR2* and its partners (*SLMAP*, *UBP1*, *CTNNA3*, *INA*, *DZANK1*, *MYPN*, *CGNL1*, *AMPD2*, and *ARHGAP24*). (B) Fluorescence in situ hybridization confirmed the break-apart signal of the *FGFR2* probe in the fusion-positive tumor tissue but not in the matched normal tissue (Left: representative images of tumor tissues, with arrows indicating *FGFR2* translocation; right: representative images of matched nontumor tissues, with circles indicating fusion-negative alleles).

To validate whether these detected fusion genes are transcribed into mRNA, these fusion transcripts were amplified from the tumor cDNA and sequenced. The mRNA sequences flanking the breakpoints were found to be identical to the consensus sequences obtained from AMP RNA sequencing; this was further confirmed by the BLAT-UCSC and Ensembl genome databases (Fig 2A). We then validated the *FGFR* fusions by using the break-apart FISH technique. For the only case with an *FGFR3* fusion, the two genes involved were too close in location to be discerned by FISH. Of the 12 cases with *FGFR2* fusions, only 5 had high-quality FFPE material for FISH analysis. Among these 5 cases, we detected tumor-specific *FGFR2* translocations in > 50% of tumor cells in 4 cases and in 5% of tumor cells in the tumor harboring the *FGFR2-UBP1* fusion (Fig 2B).

### Prevalence of *FGFR* Fusions in CCA With Different Etiologies and Anatomic Subtypes

Of note, *FGFR* fusions were strikingly enriched in non–fluke-associated CCA tumors (11.6%; 11/95) compared with fluke-associated CCA tumors (1.65%; 2/121), suggesting that *FGFR* fusions might play a crucial role in carcinogenesis of non–fluke-associated CCA, but not fluke-associated CCA.

Among the FGFR family genes, *FGFR2* fusions showed the highest frequency in our cohort. Of these, the highest number of 15.7% (8/51) was found in samples from Singapore, followed by 6.8% (3/44) in samples from Romania, and finally only 0.8% (1/121) in samples from Thailand.

Existing CCA classification systems are primarily based on anatomic location—intrahepatic CCA (ICC) or extrahepatic CCA. We observed that *FGFR* fusion-positive tumors were exclusively found in the ICC subset of tumors.

### Genomic Landscape of *FGFR* Alterations in CCA

**FIG 3:**
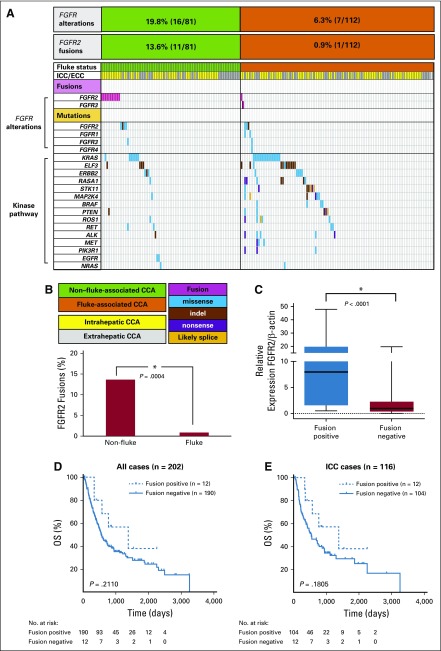
Genetic alterations of *FGFR* family genes and kinase-related genes. (A) Integration of our previously published mutational results with fusion events screened in 193 cholangiocarcinoma (CCA) cases. *FGFR* alterations mainly occur in non–fluke-associated and intrahepatic CCA. (B) *FGFR2* fusions were almost exclusively detected in non–fluke-associated CCA (11/95) compared with fluke-associated CCA (1/121; *P* = .0006). (C) Expression of *FGFR2* was analyzed by quantitative polymerase chain reaction (n = 190 cases). *FGFR2* expression in fusion-positive cases were significantly higher (n = 11) than in fusion-negative cases (n = 179; *P* < .0001). (D, E) Kaplan-Meier survival curves for overall survival (OS) stratified by *FGFR2/FGFR3* fusions in (D) all cases, and (E) intrahepatic CCA (ICC). Fusion-positive tumors showed a trend toward better OS relative to fusion-negative tumors, but this was not statistically significant. ECC, extrahepatic cholangiocarcinoma.

To comprehensively explore the genetic basis of *FGFR* alterations in CCA, we integrated the results of our *FGFR* fusion analysis with published data (n = 193 merged cases) from whole-genome and targeted sequencing.^5^ We observed 12% (23/193) of tumors harboring *FGFR* alterations (somatic mutations or fusions). Mutations in *FGFR1*, *FGFR2*, *FGFR3*, and *FGFR4* were present in 1.0%, 3.6%, 1.0%, and 0.5% of tumors. The most frequent alteration was *FGFR2* fusion (6.2%; 12/193). Notably, *FGFR2/3* fusions were mutually exclusive with somatic mutations in other kinase-related genes (*KRAS/ERBB2/BRAF/FGFR* mutations; Fig 3A).

*FGFR2* fusions were clearly enriched in non–fluke-associated CCA (13.6%; 11/81) compared with fluke-associated cases (0.9%; 1/112; *P* = .0004; Fig 3B). As *FGFR* fusion can trigger the upregulation of *FGFR* expression, we determined the expression levels of *FGFR2* in fusion-positive cases. Expression of *FGFR2* was significantly higher in tumors with *FGFR2* fusion compared with tumors without *FGFR2* fusion (*P* < .0001; Fig 3C). This result confirmed that *FGFR2* upregulation is a tumor-specific event.

In addition to the detected chimeric FGFR fusion transcripts, we further investigated the genomic somatic copy gains of FGFRs using our previously published SNP6 array data of 175 paired tumor-normal samples^5^ with ASCAT. The ASCAT analysis detected 19 copy gains of FGFRs in 15 unique CCA tumors: FGFR1 (4/175), FGFR2 (2/175), FGFR3 (6/175), and FGFR4 (7/175; Data Supplement). Of note, amplification of FGFRs was not found in our screening cohort, implying that mainly fusion and mutation of FGFRs are involved in carcinogenesis of CCA.

### Clinicopathologic Characteristics of Patients Harboring *FGFR* Fusions

Finally, we determined whether there were any associations between *FGFR2/3* fusions and clinico-pathologic characteristics (Tables 1 and 2). *FGFR* fusion-positive tumors predominantly presented in younger patients (*P* = .043, Fisher’s exact test), and were also significantly associated with the intrahepatic subtype, as well as with moderate differentiation histology (*P* = .009 and *P* = .016, respectively, Fisher’s exact test). These tumors showed a trend toward better overall survival compared with fusion-negative tumors, although this difference was not statistically significant (Figs 3D and 3E).

**TABLE 2:**
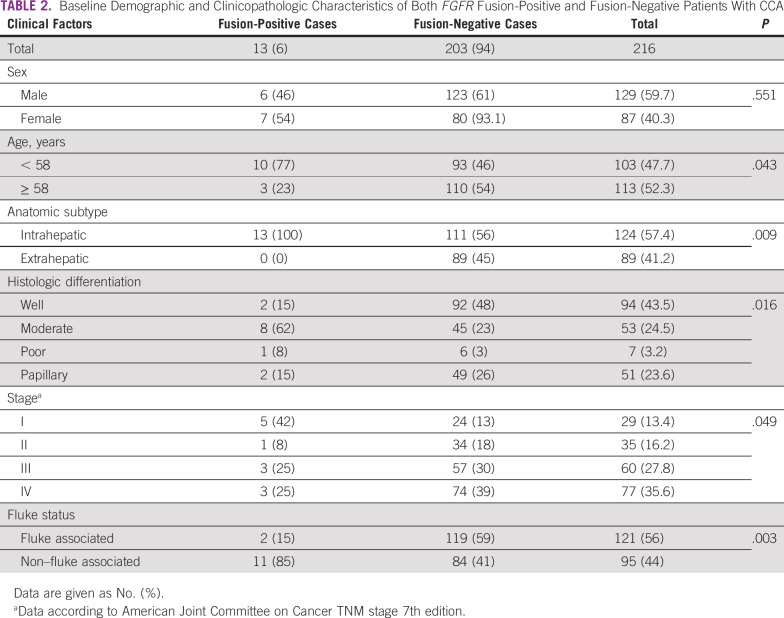
Baseline Demographic and Clinicopathologic Characteristics of Both *FGFR* Fusion-Positive and Fusion-Negative Patients With CCA

## DISCUSSION

Gene fusions involving the *FGFR* family have been implicated as oncogenic drivers in various human cancers.^24^ Tumorigenesis driven by *FGFR* fusions can be treated effectively with kinase inhibitors, highlighting the importance of detecting these gene fusions in clinical samples. Conventional cytogenetics is regarded as the gold standard method for detecting such rearrangements, but this technique is time consuming and requires a high level of expertise, making it unsuitable for the detection of many cryptic rearrangements.^25^ Complementary approaches, such as FISH and reverse transcription PCR, on the other hand, suffer from a lack of scalability, because only a few genes can be interrogated simultaneously. High-throughput sequencing technologies, such as whole-genome, whole-exome, and RNA sequencing, are currently impractical for use in clinical diagnostics because of their high cost and low efficiency. Here, we adopted a recently described targeted RNA sequencing approach, AMP, which requires low RNA input and is able to rapidly identify a broad range of gene fusions.^12^ Using this approach, we comprehensively analyzed 216 CCA tumors of different geographical and etiological origins, namely the endemic fluke-associated CCA from the northeast of Thailand compared with non–fluke-associated cases from Singapore and Romania. Overall, we identified *FGFR2/FGFR3* fusions in 6% of CCA tumors. We particularly want to focus on *FGFR2* fusions, which formed the molecular basis for two previously reported clinical trials on FGFR inhibitors in CCA, which both showed promising clinical responses. When examining the *FGFR2* fusions in our study, we observed a distinction in frequency between fluke-associated cases (1/121) versus non–fluke-associated cases (11/95; *P* = .0006), indicating that *FGFR* fusions were almost exclusively detected in non–fluke-associated CCA. These results serve as yet another example of what we previously reported regarding the distinct mutation pattern and frequency among CCA of these different etiologies. For example, *TP53* mutations are found in almost half of the fluke-associated CCA but only in approximately 10% of non–fluke-associated CCA. On the other hand, *BAP1* and *IDH* mutations are found in approximately 20%-25% of non–fluke-associated CCA but only 2%-3% of fluke-associated CCA.^4^

In keeping with previous reports,^6,9,11,17^ *FGFR2* fusions occurred exclusively in intrahepatic CCA, suggesting the existence of subtype- and etiology-specific differences in tumorigenesis. Clearly, *FGFR2* fusions outnumbered *FGFR3* fusions in CCA as, out of the 13 fusions detected, 12 were *FGFR2* fusions. Only a single *FGFR3-TACC3* fusion previously reported in other cancers was identified.^10,19^ In addition, all *FGFR* fusions were mutually exclusive with *KRAS/ERBB2/BRAF/FGFR* mutations, suggesting that these alterations are driver events, further highlighting a potential therapeutic approach for these CCA tumors. Besides *FGFR* fusions, we also examined for *FGFR* amplification and mutation. We did not detect any amplifications on the basis of ASCAT analysis. In the phase II study on BGJ398 in advanced CCA, only 1 of 3 cases with *FGFR2* amplification showed a reduction in tumor size (by 27%).^15^ Similarly, in the same trial, only 1 of 8 cases with *FGFR2* mutation showed reduction in size (by 23%). Taken together, whether *FGFR* amplification and mutation correlate with response to FGFR inhibitors needs to be further explored.

Collectively, our findings illustrate the importance of conducting cancer genomic studies in diverse populations so as to enable the molecular dissection of specific cancer types for translational benefit. For example, in this scenario, the same cancer in fluke-endemic Thailand not only suffers from worse prognosis^1^ it unfortunately also presents with a profound lack of actionable targets, therefore representing an area of unmet clinical need that requires novel therapeutic strategies.

In summary, we comprehensively analyzed fusions involving *FGFR* family genes using a targeted RNA sequencing approach. By comparing between CCA tumors with different etiologies, we found that *FGFR2* fusions were almost exclusively associated with non–fluke-associated CCA. For endemic fluke-associated CCA, which carries a poorer prognosis compared with its non–fluke-associated counterpart,^5^ there is an urgent need to identify specific targets that are druggable. The current study also highlights the importance to conduct genomic and other studies on cancer in diverse populations.
